# 2-(4-Bromo­anilino)-6-(4-chloro­phen­yl)-5-meth­oxy­carbonyl-4-methyl-3,6-dihydro­pyrimidin-1-ium chloride

**DOI:** 10.1107/S1600536813006296

**Published:** 2013-03-09

**Authors:** K. N. Venugopala, Susanta K. Nayak, Bharti Odhav

**Affiliations:** a Department of Biotechnology and Food Technology, Durban University of Technology, Durban 4001, South Africa; b Equipe Chimie du Solide et Matériaux, UMR 6226 Institut des Sciences, Université de Rennes 1, Campus de Beaulieu, Avenue du Général Leclerc, 35042 Rennes cedex, France

## Abstract

In the title molecular salt, C_19_H_18_BrClN_3_O_2_^+^·Cl^−^, the dihedral angles between the pyrimidine ring and the chlorobenzene and bromobenzene rings are 72.4 (2) and 45.5 (2)°, respectively. The dihedral angle between the chlorobenzene and bromobenzene rings is 27.5 (2)°. The conformation of the mol­ecule is stabilized by an intra­molecular C—H⋯O inter­action. In the crystal, the anion and cation are linked by an N—H⋯Cl hydrogen bond. Pairs of weak C—H⋯O and C—H⋯Cl hydrogen bonds form inversion dimers. Further N—H⋯Cl hydrogen bonds form *R*_2_^1^(6) motifs and link the dimers into chains along [101]. Br⋯Cl short contacts [3.482 (2) Å] inter­link the hydrogen-bonded chains along the *b*-axis direction.

## Related literature

For a study of chloride salts of dihydro­pyrimidine derivatives and their anti-tubercular activity, see: Venugopala, Nayak, Pillay *et al.* (2012 ▶). For the crystal structures of dihydro­pyrimidine derivatives, see: Venugopala, Nayak & Odhav (2012 ▶). For hydrogen-bond motifs, see: Bernstein *et al.* (1995 ▶).
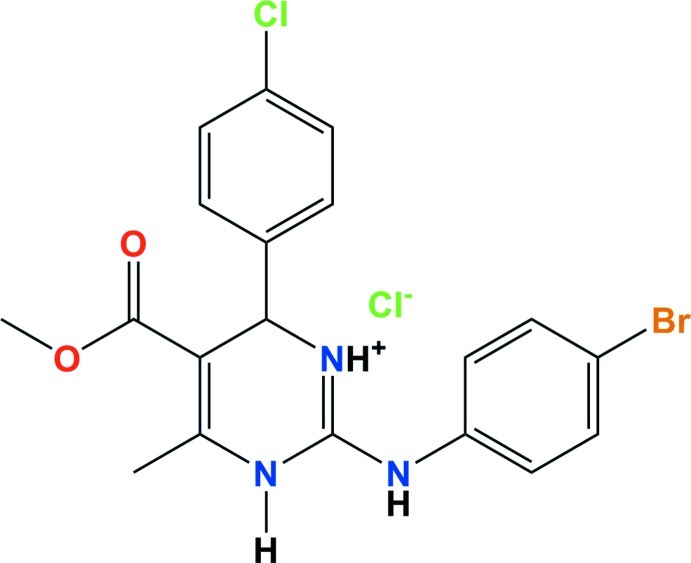


## Experimental

### 

#### Crystal data

C_19_H_18_BrClN_3_O_2_^+^·Cl^−^
*M* *_r_* = 471.17
Monoclinic, 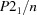

*a* = 13.2691 (15) Å
*b* = 11.0965 (12) Å
*c* = 14.9545 (17) Å
β = 114.181 (3)°
*V* = 2008.7 (4) Å^3^
*Z* = 4
Mo *K*α radiation
μ = 2.33 mm^−1^
*T* = 100 K
0.08 × 0.05 × 0.03 mm

#### Data collection

Bruker Kappa DUO APEXII diffractometer
Absorption correction: multi-scan (*SADABS*; Bruker, 2008 ▶) *T*_min_ = 0.835, *T*_max_ = 0.933
10242 measured reflections
3919 independent reflections
2511 reflections with *I* > 2σ(*I*)
*R*_int_ = 0.067

#### Refinement

*R*[*F* ^2^ > 2σ(*F* ^2^)] = 0.044
*wR*(*F* ^2^) = 0.096
*S* = 0.95
3919 reflections
246 parameters
H-atom parameters constrained
Δρ_max_ = 0.42 e Å^−3^
Δρ_min_ = −0.40 e Å^−3^

### 

Data collection: *APEX2* (Bruker, 2008 ▶); cell refinement: *SAINT* (Bruker, 2008 ▶); data reduction: *SAINT*; program(s) used to solve structure: *SHELXS97* (Sheldrick, 2008 ▶); program(s) used to refine structure: *SHELXL97* (Sheldrick, 2008 ▶); molecular graphics: *ORTEP-3 for Windows* (Farrugia, 2012 ▶) and *Mercury* (Macrae *et al.*, 2008 ▶); software used to prepare material for publication: *PLATON* (Spek, 2009 ▶) and *PARST* (Nardelli, 1995 ▶).

## Supplementary Material

Crystal structure: contains datablock(s) global, I. DOI: 10.1107/S1600536813006296/pv2621sup1.cif

Structure factors: contains datablock(s) I. DOI: 10.1107/S1600536813006296/pv2621Isup2.hkl

Supplementary material file. DOI: 10.1107/S1600536813006296/pv2621Isup3.cml

Additional supplementary materials:  crystallographic information; 3D view; checkCIF report

## Figures and Tables

**Table 1:** Hydrogen-bond geometry (Å, °)

*D*—H⋯*A*	*D*—H	H⋯*A*	*D*⋯*A*	*D*—H⋯*A*
N1—H1⋯Cl2	0.88	2.34	3.136 (3)	151
N2—H2⋯Cl2^i^	0.88	2.41	3.179 (3)	146
N3—H3⋯Cl2^i^	0.88	2.39	3.191 (3)	151
C5—H5*A*⋯O2	0.98	2.22	2.897 (5)	125
C15—H15⋯O2^ii^	0.95	2.42	3.197 (5)	139
C18—H18⋯Cl2^iii^	0.95	2.81	3.702 (4)	156

Symmetry codes: (i) 
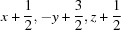
; (ii) 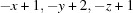; (iii) 
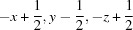
.

**Fig. 1.**
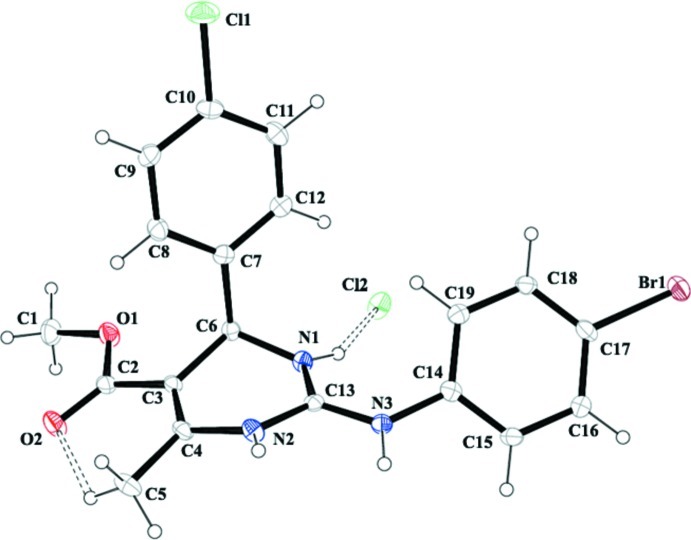
A view of the title compound with the atom numbering scheme and displacement ellipsoids for non-H atoms drawn at the 50% probability level. The intramolecular interactions are shown as dashed lines.

**Fig. 2.**
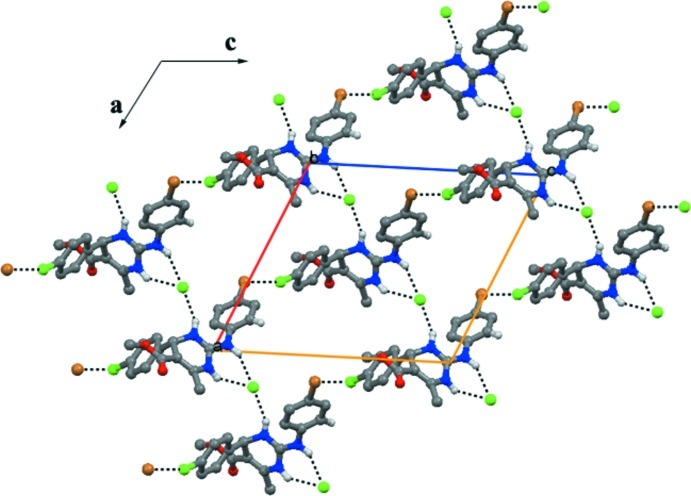
Intermolecular N—H···Cl hydrogen-bonding interactions form infinite chains which are linked by Br···Cl short contacts along the b axis.

## supplementary crystallographic information

### Comment

We have recently reported that the chloride salts of dihydropyrimidine derivatives exhibit anti-tubercular activity (Venugopala, Nayak, Pillay *et al.* 2012). In continuation of our work in this field and on the crystal structures of dihydropyrimidine derivatives (Venugopala, Nayak & Odhav, 2012), we now report in this article, the crystal structure of the title compound.

The bond distances and angles in the title compound (Fig. 1) agree very well with the corresponding bond distances and angles reported in a closely related compound (Venugopala, Nayak &Odhav, 2012).The dihedral angles between the planes of 4-chlorophenyl and 4-bromophenyl rings with the plane of the six-membered pyrimidine ring are 72.4 (2)° and 45.5 (2)°, respectively. The conformation of the title molecule is stabilized by intramolecular C5—H5···O2 interactions. The crystal structure is stabilized by the N—H···Cl hydrogen bonds and further consolidated by weak C—H···O and C—H···Cl hydrogen bonding interactions (Table 1 & Fig. 2).

In the crystal structure, Cl2 is hydrogen bonded to N2 and N3 forming six membered rings in R_2_^1^(6) motif (Bernstein *et al.*,1995). This Cl2 is also involved in a hydrogen bond (C18—H18···Cl2) with a molecule lying about an inversion center thus resulting in dimers. In addition, N1—H1···Cl2 interactions link the dimers into chains in the (1 0 1) direction. C15—H15···O2 interactions also result in macrocyclic rings about inversion centers resulting in dimers. Finally, a Br1···Cl1^*^ short contact (3.482 (2) Å, symmetry code ^*^: *x* - 1/2, -*y* + 1/2, *z* + 1/2) interlinks the hydrogen bonded chains along the *b* axis.

### Experimental

A mixture of methyl-2-chloro-4-(4-chlorophenyl)-6-methyl-1,4- dihydropyrimidine-5-carboxylate (1 mmol) and 4-bromoaniline (1 mmol) in 2-propanol (5 mL) was refluxed for 16 h. The reaction was monitored by TLC. The reaction medium was cooled to room temperature and the product was filtered, washed with cold 2-propanol and dried to obtain the crude product. The product was purified by recrystallization using ethanol to yield 66% yield of product which was pale yellow amorphous solid (m.p. 500 (2) K). Crystals suitable for single-crystal X-ray analysis were obtained using acetone as a solvent using slow evaporation at room temperature.

### Refinement

All H atoms were positioned geometrically with N—H = 0.88 Å, C—H = 0.95–1.00 Å and refined using a riding model with *U*_iso_(H) = 1.2 *U*_eq_(C/N)except for the methyl group where *U*_iso_(H) = 1.5 *U*_eq_(C).

### Crystal data

**Table:**

C_19_H_18_BrClN_3_O_2_^+^·Cl^−^	*F*(000) = 952
*M**_r_* = 471.17	*D*_x_ = 1.558 Mg m^−^^3^
Monoclinic, *P*2_1_/*n*	Melting point: 500(2) K
Hall symbol: -P 2yn	Mo *K*α radiation, λ = 0.71073 Å
*a* = 13.2691 (15) Å	Cell parameters from 650 reflections
*b* = 11.0965 (12) Å	θ = 1.7–27.9°
*c* = 14.9545 (17) Å	µ = 2.33 mm^−^^1^
β = 114.181 (3)°	*T* = 100 K
*V* = 2008.7 (4) Å^3^	Block, colorless
*Z* = 4	0.08 × 0.05 × 0.03 mm

### Data collection

**Table:**

Bruker Kappa DUO APEXII diffractometer	3919 independent reflections
Radiation source: fine-focus sealed tube	2511 reflections with *I* > 2σ(*I*)
Graphite monochromator	*R*_int_ = 0.067
0.5° φ scans and ω scans	θ_max_ = 26.0°, θ_min_ = 1.7°
Absorption correction: multi-scan (*SADABS*; Bruker, 2008)	*h* = −16→16
*T*_min_ = 0.835, *T*_max_ = 0.933	*k* = −13→13
10242 measured reflections	*l* = −18→16

### Refinement

**Table:**

Refinement on *F*^2^	Primary atom site location: structure-invariant direct methods
Least-squares matrix: full	Secondary atom site location: difference Fourier map
*R*[*F*^2^ > 2σ(*F*^2^)] = 0.044	Hydrogen site location: inferred from neighbouring sites
*wR*(*F*^2^) = 0.096	H-atom parameters constrained
*S* = 0.95	*w* = 1/[σ^2^(*F*_o_^2^) + (0.0403*P*)^2^] where *P* = (*F*_o_^2^ + 2*F*_c_^2^)/3
3919 reflections	(Δ/σ)_max_ < 0.001
246 parameters	Δρ_max_ = 0.42 e Å^−^^3^
0 restraints	Δρ_min_ = −0.40 e Å^−^^3^

### Special details

**Table:**

Geometry. All s.u.'s (except the s.u. in the dihedral angle between two l.s. planes) are estimated using the full covariance matrix. The cell s.u.'s are taken into account individually in the estimation of s.u.'s in distances, angles and torsion angles; correlations between s.u.'s in cell parameters are only used when they are defined by crystal symmetry. An approximate (isotropic) treatment of cell s.u.'s is used for estimating s.u.'s involving l.s. planes.
Refinement. Refinement of *F*^2^ against ALL reflections. The weighted *R*-factor *wR* and goodness of fit *S* are based on *F*^2^, conventional *R*-factors *R* are based on *F*, with *F* set to zero for negative *F*^2^. The threshold expression of *F*^2^ > 2σ(*F*^2^) is used only for calculating *R*-factors(gt) *etc*. and is not relevant to the choice of reflections for refinement. *R*-factors based on *F*^2^ are statistically about twice as large as those based on *F*, and *R*- factors based on ALL data will be even larger.

### Fractional atomic coordinates and isotropic or equivalent isotropic displacement parameters (Å^2^)

**Table:**

	*x*	*y*	*z*	*U*_iso_*/*U*_eq_	
Br1	0.13690 (4)	0.30575 (4)	0.48412 (3)	0.03906 (15)	
Cl1	0.62679 (9)	0.43238 (11)	0.13181 (9)	0.0497 (3)	
Cl2	0.18134 (7)	0.73567 (10)	0.24229 (7)	0.0366 (3)	
O1	0.4928 (2)	1.0157 (2)	0.21182 (19)	0.0336 (7)	
O2	0.6208 (2)	1.1174 (3)	0.3359 (2)	0.0391 (7)	
N1	0.4274 (2)	0.7807 (3)	0.3884 (2)	0.0220 (7)	
H1	0.3580	0.7579	0.3678	0.026*	
N2	0.5907 (2)	0.8308 (3)	0.5160 (2)	0.0244 (7)	
H2	0.6422	0.8109	0.5736	0.029*	
N3	0.4682 (2)	0.7087 (3)	0.5462 (2)	0.0236 (7)	
H3	0.5075	0.7251	0.6085	0.028*	
C1	0.4941 (4)	1.1104 (4)	0.1460 (3)	0.0420 (11)	
H1A	0.5703	1.1363	0.1632	0.063*	
H1B	0.4503	1.1788	0.1515	0.063*	
H1C	0.4624	1.0801	0.0785	0.063*	
C2	0.5598 (3)	1.0312 (3)	0.3065 (3)	0.0266 (9)	
C3	0.5487 (3)	0.9312 (3)	0.3668 (3)	0.0213 (8)	
C4	0.6119 (3)	0.9232 (3)	0.4638 (3)	0.0239 (9)	
C5	0.7060 (3)	1.0023 (3)	0.5265 (3)	0.0320 (10)	
H5A	0.6981	1.0819	0.4961	0.048*	
H5B	0.7760	0.9660	0.5329	0.048*	
H5C	0.7056	1.0105	0.5916	0.048*	
C6	0.4680 (3)	0.8309 (3)	0.3176 (3)	0.0217 (8)	
H6	0.4033	0.8674	0.2625	0.026*	
C7	0.5126 (3)	0.7297 (3)	0.2746 (2)	0.0232 (8)	
C8	0.6080 (3)	0.7427 (4)	0.2584 (3)	0.0282 (9)	
H8	0.6504	0.8145	0.2791	0.034*	
C9	0.6427 (3)	0.6536 (4)	0.2129 (3)	0.0308 (9)	
H9	0.7070	0.6643	0.2008	0.037*	
C10	0.5819 (3)	0.5490 (4)	0.1855 (3)	0.0308 (10)	
C11	0.4875 (3)	0.5316 (4)	0.2014 (3)	0.0305 (10)	
H11	0.4475	0.4581	0.1833	0.037*	
C12	0.4528 (3)	0.6233 (4)	0.2441 (3)	0.0268 (9)	
H12	0.3863	0.6137	0.2529	0.032*	
C13	0.4936 (3)	0.7697 (3)	0.4817 (3)	0.0202 (8)	
C14	0.3842 (3)	0.6193 (3)	0.5253 (3)	0.0230 (8)	
C15	0.3303 (3)	0.6112 (4)	0.5866 (3)	0.0284 (9)	
H15	0.3441	0.6692	0.6370	0.034*	
C16	0.2553 (3)	0.5180 (4)	0.5747 (3)	0.0301 (9)	
H16	0.2184	0.5112	0.6170	0.036*	
C17	0.2358 (3)	0.4357 (3)	0.5001 (3)	0.0277 (9)	
C18	0.2874 (3)	0.4450 (3)	0.4374 (3)	0.0246 (9)	
H18	0.2711	0.3889	0.3853	0.029*	
C19	0.3625 (3)	0.5356 (3)	0.4501 (3)	0.0237 (8)	
H19	0.3996	0.5412	0.4078	0.028*	

### Atomic displacement parameters (Å^2^)

**Table:**

	*U*^11^	*U*^22^	*U*^33^	*U*^12^	*U*^13^	*U*^23^
Br1	0.0491 (3)	0.0302 (2)	0.0386 (3)	−0.0145 (2)	0.0188 (2)	−0.0051 (2)
Cl1	0.0502 (7)	0.0405 (7)	0.0635 (8)	0.0083 (5)	0.0285 (6)	−0.0154 (6)
Cl2	0.0226 (5)	0.0571 (7)	0.0259 (5)	−0.0006 (5)	0.0058 (4)	0.0065 (5)
O1	0.0401 (16)	0.0293 (17)	0.0292 (16)	−0.0082 (13)	0.0117 (13)	0.0027 (12)
O2	0.0433 (17)	0.0265 (16)	0.0440 (18)	−0.0114 (14)	0.0144 (14)	−0.0019 (14)
N1	0.0136 (14)	0.0266 (19)	0.0221 (17)	−0.0004 (12)	0.0036 (13)	0.0000 (13)
N2	0.0169 (16)	0.0249 (19)	0.0239 (17)	−0.0012 (13)	0.0007 (13)	0.0002 (13)
N3	0.0243 (16)	0.0268 (19)	0.0173 (16)	−0.0040 (14)	0.0060 (13)	−0.0026 (14)
C1	0.052 (3)	0.040 (3)	0.038 (3)	−0.009 (2)	0.022 (2)	0.007 (2)
C2	0.027 (2)	0.023 (2)	0.034 (2)	−0.0016 (17)	0.0166 (19)	−0.0060 (18)
C3	0.0227 (19)	0.017 (2)	0.026 (2)	−0.0006 (15)	0.0112 (17)	−0.0029 (16)
C4	0.022 (2)	0.017 (2)	0.032 (2)	−0.0007 (15)	0.0109 (18)	−0.0044 (16)
C5	0.026 (2)	0.024 (2)	0.038 (2)	−0.0018 (17)	0.0033 (18)	−0.0081 (19)
C6	0.0198 (18)	0.023 (2)	0.0206 (19)	−0.0013 (15)	0.0063 (16)	0.0009 (15)
C7	0.026 (2)	0.023 (2)	0.0172 (19)	0.0028 (16)	0.0063 (16)	0.0027 (15)
C8	0.024 (2)	0.028 (2)	0.031 (2)	−0.0029 (17)	0.0097 (17)	−0.0013 (18)
C9	0.028 (2)	0.032 (2)	0.038 (2)	0.0020 (18)	0.0194 (19)	−0.0010 (19)
C10	0.033 (2)	0.025 (2)	0.032 (2)	0.0083 (18)	0.0110 (19)	−0.0031 (18)
C11	0.028 (2)	0.026 (2)	0.031 (2)	−0.0031 (17)	0.0042 (18)	−0.0004 (18)
C12	0.024 (2)	0.030 (2)	0.025 (2)	0.0034 (17)	0.0092 (17)	0.0019 (18)
C13	0.0185 (18)	0.019 (2)	0.025 (2)	0.0029 (15)	0.0107 (17)	−0.0035 (16)
C14	0.025 (2)	0.019 (2)	0.021 (2)	0.0047 (15)	0.0052 (17)	0.0040 (16)
C15	0.038 (2)	0.024 (2)	0.024 (2)	−0.0020 (18)	0.0140 (19)	−0.0055 (17)
C16	0.037 (2)	0.030 (2)	0.029 (2)	−0.0050 (18)	0.0176 (19)	−0.0007 (18)
C17	0.034 (2)	0.018 (2)	0.027 (2)	−0.0033 (16)	0.0074 (18)	−0.0001 (17)
C18	0.030 (2)	0.022 (2)	0.021 (2)	−0.0001 (16)	0.0093 (17)	−0.0026 (16)
C19	0.025 (2)	0.022 (2)	0.024 (2)	0.0063 (16)	0.0098 (17)	0.0028 (17)

### Geometric parameters (Å, º)

**Table:**

Br1—C17	1.898 (4)	C5—H5C	0.9800
Cl1—C10	1.751 (4)	C6—C7	1.529 (5)
O1—C2	1.339 (4)	C6—H6	1.0000
O1—C1	1.445 (5)	C7—C8	1.390 (5)
O2—C2	1.214 (4)	C7—C12	1.391 (5)
N1—C13	1.315 (4)	C8—C9	1.382 (5)
N1—C6	1.479 (4)	C8—H8	0.9500
N1—H1	0.8800	C9—C10	1.377 (5)
N2—C13	1.357 (4)	C9—H9	0.9500
N2—C4	1.386 (5)	C10—C11	1.381 (5)
N2—H2	0.8800	C11—C12	1.377 (5)
N3—C13	1.330 (4)	C11—H11	0.9500
N3—C14	1.428 (4)	C12—H12	0.9500
N3—H3	0.8800	C14—C15	1.377 (5)
C1—H1A	0.9800	C14—C19	1.395 (5)
C1—H1B	0.9800	C15—C16	1.395 (5)
C1—H1C	0.9800	C15—H15	0.9500
C2—C3	1.475 (5)	C16—C17	1.381 (5)
C3—C4	1.349 (5)	C16—H16	0.9500
C3—C6	1.510 (5)	C17—C18	1.373 (5)
C4—C5	1.500 (5)	C18—C19	1.374 (5)
C5—H5A	0.9800	C18—H18	0.9500
C5—H5B	0.9800	C19—H19	0.9500
			
C2—O1—C1	116.1 (3)	C8—C7—C6	122.3 (3)
C13—N1—C6	120.9 (3)	C12—C7—C6	119.6 (3)
C13—N1—H1	119.6	C9—C8—C7	121.5 (4)
C6—N1—H1	119.6	C9—C8—H8	119.3
C13—N2—C4	122.4 (3)	C7—C8—H8	119.3
C13—N2—H2	118.8	C10—C9—C8	118.5 (4)
C4—N2—H2	118.8	C10—C9—H9	120.7
C13—N3—C14	127.0 (3)	C8—C9—H9	120.7
C13—N3—H3	116.5	C9—C10—C11	121.9 (4)
C14—N3—H3	116.5	C9—C10—Cl1	119.5 (3)
O1—C1—H1A	109.5	C11—C10—Cl1	118.6 (3)
O1—C1—H1B	109.5	C12—C11—C10	118.4 (4)
H1A—C1—H1B	109.5	C12—C11—H11	120.8
O1—C1—H1C	109.5	C10—C11—H11	120.8
H1A—C1—H1C	109.5	C11—C12—C7	121.6 (4)
H1B—C1—H1C	109.5	C11—C12—H12	119.2
O2—C2—O1	122.6 (4)	C7—C12—H12	119.2
O2—C2—C3	126.2 (4)	N1—C13—N3	124.0 (3)
O1—C2—C3	111.1 (3)	N1—C13—N2	118.3 (3)
C4—C3—C2	122.2 (3)	N3—C13—N2	117.6 (3)
C4—C3—C6	118.7 (3)	C15—C14—C19	120.1 (3)
C2—C3—C6	119.0 (3)	C15—C14—N3	118.2 (3)
C3—C4—N2	118.3 (3)	C19—C14—N3	121.6 (3)
C3—C4—C5	128.8 (4)	C14—C15—C16	120.1 (4)
N2—C4—C5	112.9 (3)	C14—C15—H15	120.0
C4—C5—H5A	109.5	C16—C15—H15	120.0
C4—C5—H5B	109.5	C17—C16—C15	118.8 (4)
H5A—C5—H5B	109.5	C17—C16—H16	120.6
C4—C5—H5C	109.5	C15—C16—H16	120.6
H5A—C5—H5C	109.5	C18—C17—C16	121.4 (4)
H5B—C5—H5C	109.5	C18—C17—Br1	118.9 (3)
N1—C6—C3	108.8 (3)	C16—C17—Br1	119.7 (3)
N1—C6—C7	110.0 (3)	C17—C18—C19	119.8 (3)
C3—C6—C7	115.3 (3)	C17—C18—H18	120.1
N1—C6—H6	107.5	C19—C18—H18	120.1
C3—C6—H6	107.5	C18—C19—C14	119.9 (3)
C7—C6—H6	107.5	C18—C19—H19	120.1
C8—C7—C12	118.0 (4)	C14—C19—H19	120.1
			
C1—O1—C2—O2	−2.1 (5)	C8—C9—C10—C11	0.6 (6)
C1—O1—C2—C3	178.1 (3)	C8—C9—C10—Cl1	−177.6 (3)
O2—C2—C3—C4	−3.4 (6)	C9—C10—C11—C12	1.4 (6)
O1—C2—C3—C4	176.4 (3)	Cl1—C10—C11—C12	179.7 (3)
O2—C2—C3—C6	179.9 (4)	C10—C11—C12—C7	−2.6 (6)
O1—C2—C3—C6	−0.3 (5)	C8—C7—C12—C11	1.6 (5)
C2—C3—C4—N2	175.7 (3)	C6—C7—C12—C11	177.4 (3)
C6—C3—C4—N2	−7.6 (5)	C6—N1—C13—N3	−168.9 (3)
C2—C3—C4—C5	−4.6 (6)	C6—N1—C13—N2	15.6 (5)
C6—C3—C4—C5	172.2 (3)	C14—N3—C13—N1	18.7 (6)
C13—N2—C4—C3	−18.5 (5)	C14—N3—C13—N2	−165.8 (3)
C13—N2—C4—C5	161.7 (3)	C4—N2—C13—N1	14.7 (5)
C13—N1—C6—C3	−37.3 (4)	C4—N2—C13—N3	−161.0 (3)
C13—N1—C6—C7	89.8 (4)	C13—N3—C14—C15	−146.4 (4)
C4—C3—C6—N1	32.5 (4)	C13—N3—C14—C19	38.7 (5)
C2—C3—C6—N1	−150.7 (3)	C19—C14—C15—C16	1.2 (5)
C4—C3—C6—C7	−91.6 (4)	N3—C14—C15—C16	−173.8 (3)
C2—C3—C6—C7	85.3 (4)	C14—C15—C16—C17	−0.7 (6)
N1—C6—C7—C8	−139.6 (3)	C15—C16—C17—C18	−0.9 (6)
C3—C6—C7—C8	−16.2 (5)	C15—C16—C17—Br1	178.5 (3)
N1—C6—C7—C12	44.8 (4)	C16—C17—C18—C19	1.9 (6)
C3—C6—C7—C12	168.2 (3)	Br1—C17—C18—C19	−177.4 (3)
C12—C7—C8—C9	0.5 (5)	C17—C18—C19—C14	−1.4 (5)
C6—C7—C8—C9	−175.2 (3)	C15—C14—C19—C18	−0.1 (5)
C7—C8—C9—C10	−1.5 (6)	N3—C14—C19—C18	174.7 (3)

### Hydrogen-bond geometry (Å, º)

**Table:**

*D*—H···*A*	*D*—H	H···*A*	*D*···*A*	*D*—H···*A*
N1—H1···Cl2	0.88	2.34	3.136 (3)	151
N2—H2···Cl2^i^	0.88	2.41	3.179 (3)	146
N3—H3···Cl2^i^	0.88	2.39	3.191 (3)	151
C5—H5*A*···O2	0.98	2.22	2.897 (5)	125
C15—H15···O2^ii^	0.95	2.42	3.197 (5)	139
C18—H18···Cl2^iii^	0.95	2.81	3.702 (4)	156

Symmetry codes: (i) *x*+1/2, −*y*+3/2, *z*+1/2; (ii) −*x*+1, −*y*+2, −*z*+1; (iii) −*x*+1/2, *y*−1/2, −*z*+1/2.
